# Sterilization protocols and the effect of plant growth regulators on callus induction and secondary metabolites production in in vitro cultures *Melia azedarach* L.

**DOI:** 10.1186/s13568-022-01343-8

**Published:** 2022-01-10

**Authors:** Fatemeh Ahmadpoor, Nasser Zare, Rasool Asghari, Parisa Sheikhzadeh

**Affiliations:** Department of Plant Production and Genetics, Faculty of Agriculture and Natural Resources, University of Mohaghegh Ardabili, P.O. Box 179, Ardabil, Iran

**Keywords:** Disinfection, Hydrogen peroxide, Kaempferol, *Melia azedarach* L., Quercetin, Rutin

## Abstract

*Melia azedarach* L. is a valuable source of antioxidants and secondary metabolites. This study is a first extensive report about the effect of different serialization protocols and plant growth regulators (PGRs) on explant disinfection efficiency, callus induction and secondary metabolites production and accumulation in callus cultures of *M. azedarach* L. In this regard, the effect of plant growth regulators on callus induction and secondary metabolites production were examined. In addition, different sterilization agents were evaluated for disinfection of chinaberry leaf explants. The results showed that the lowest percentage of explant contamination and browning with the highest percentage of callus induction and callus growth obtained with explants pretreated with benomyl (2 g/L) for 2 h and sterilized with 7% H_2_O_2_ for 10 min and NaOCl 2% (without pH adjustment) for 12 min. Although adjusting the pH of NaOCl to pH  = 7 and 10 significantly reduced the microbial contamination and increased the percentage of contamination-free cultures of *M. azedarach* L., adversely influenced the explant viability and callus induction and growth. The highest percentage of callus induction obtained on the MS medium containing 3 mg/L NAA/2,4-D and 1 or 3 mg/L Kin/BAP, and the highest callus yield (1804.833 mg/explant) belonged to the MS medium supplemented with 5 mg/L 2,4-D and 5 mg/L Kin. The callus cultures grown on the MS medium supplemented with 3 mg/L NAA and 1 mg/L Kin produced the highest amount of Quercetin (2.06 mg/g fresh weight), Rutin (5.56 mg/g fresh weight) and Kaempferol (1.84 mg/g fresh weight).

**Supplementary Information:**

The online version contains supplementary material available at 10.1186/s13568-022-01343-8.

## Introduction

*Melia azedarach* L. (known as Chinaberry) is one of the most valuable pharmaceutical plants. It has therapeutic properties such as antihyperglycemic, anticarcinogenic, anti-inflammatory, antiviral, insecticidal and antioxidant effects. A large numbers of secondary metabolites such as alkaloids, phenolic compounds, and vitamins have been recognized in *M. azedarach* L. (Yalla et al. 2021; Coria et al. 2008). The success of a plant tissue culture relies on many factors, such as culture medium composition, particularly plant growth regulators (PGRs), age and other physiological aspects of donor plant (Zare et al. 2009; Asghari Zakaria et al. 2011).

In vitro fungal and bacterial contaminations are also one of the most important limiting factors, particularly in woody plants. In order to overcome fungal and bacterial contaminations in the disinfection stage, different surface sterilizing agents including sodium and calcium hypochlorite, ethanol, mercuric chloride, silver nitrate, antibiotics, and fungicide were used in laboratories worldwide. Nevertheless, the efficiency of these treatments is low in woody plants, or some of them are very toxic to plant tissues. Furthermore, the efficiency of sterilization methods depends on different parameters, such as the physiological state of the mother plant, the size, age and type of the explant, the type and concentration of sterilization agent, and exposure duration (Teixeira da Silva et al. 2015). So, it is difficult to present a standard disinfection protocol for all plant species and explants. Therefore, the sterilization procedure should be optimized for any species to gain the maximum sterilized viable explants. Assareh and Sardabi (2005) reported that among calcium and sodium hypochlorite and mercuric chloride agents used for surface sterilization of *Ziziphus spina*-christi explants, Ca(OCl)_2_ (5% for 20 min) was the most efficient in explants sterilization. In contrast, in Ghaffoor et al. (2003) studies, NaOCl was found to be efficient for removing the contaminations in *Solanum tuberosum* L. The reports on tissue culture of woody plants indicate that surface disinfection was not efficient to produce clean cultures, and always is accompanied with a high level of bacterial and fungal contamination (Pence 2005).

Biotechnological techniques, especially plant cell and tissue culture, play an essential role in exploring alternative approaches for the production of desirable therapeutic compounds from plants (Farjaminezhad et al. 2013) and environmental adaptation (Farjaminezhad and Garoosi 2021). The callus induction and establishment of suspension cultures are two basic techniques, which have been used for production of wide range of phytochemicals, including therapeutic and antioxidant compounds (Colgecen et al. 2011). Flavonoids are a large group of phenolic compounds, which are generally found in nearly all plant species. Flavonoids are known as potential metal chelators and antioxidants. Several therapeutic and disease inhibition properties of flavonoids have been reported by researchers, which make them interesting nutraceuticals in human nutrition and healthcare (Prochazkova et al. 2011). There are reports about the production of flavonoids using different biotechnological methods, including callus and cell suspension cultures (Haida et al. 2019). So, the present study was aimed to the optimize the sterilization method for *M. azedarach* L. leaf explants using different sterilization agents such as H_2_O_2_ and NaOCl, and pH adjustment of NaOCl. Moreover, the effects of different factors on callus induction from leaf explants of *M. azedarach* L. and production of secondary metabolites including rutin, quercetin and kaempferol in callus cultures was investigated.

## Materials and methods

### Explant preparation and sterilization

In this study, young leaves of Chinaberry (*M. azedarach* L.) were prepared from the Qazvin province [36°19′ N 50°00′ E and 1287 m above sea level (m a.s.l.)], Iran. The young leaves were pretreated with a soft commercial detergent and washed for 30 min. The leaf explants were sterilized using different protocols as described below.

### ***Experiment 1: the effects of different concentrations of H***_***2***_***O***_***2***_*** and NaOCl, and pH adjustment of NaOCl on the sterilization and growth of explants***

The leaf explants were dipped in benomyl (C_14_H_18_N_4_O_3_) (a systemic fungicide) solution (3 g/L) for 2 h, and then treated with 5% or 7% (v/v) H_2_O_2_ for 10 or 15 min, then soaked in 70% (v/v) ethanol for 20 s, and finally soaked in 2% (w/v) NaOCl with different pH (pH  = 7 or 10 or without pH adjustment) containing 2 drops of Tween-20 for 12 min (Additional file 1: Table S1). After that, the leaves were rinsed with sterile distilled water (SDW) for three times and then sliced into small fragments (0.5 cm squares), and cultured on MS (Murashige and Skoog 1962) medium supplemented with different concentrations of auxins [α-naphthalene acetic acid (NAA) or 2,4-Dichlorophenoxyacetic acid (2,4-D) at 3 and 5 mg/L concentrations] and cytokinins [6-benzylaminopurine (BAP) or Kinetin (Kin) at 1, 3 and 5 mg/L concentrations].

Common disinfection method including sterilization with 70% (v/v) ethanol for 20 s + 2% (w/v) NaOCl without pH adjustment for 12 min considered as control (Additional file 1: Table S1).

### Experiment 2: the effect of benomyl inclusion in culture medium on the contaminations and growth of the explants

The leaf explants were surface sterilized using 5% (v/v) H_2_O_2_ for 10 min, 70% (v/v) ethanol for 20 s and 2% (w/v) NaOCl with different pH [pH  = 10 or without pH adjustment (pH  ≥ 12)] plus Tween-20 for 12 min (Additional file 1: Table S2). Finally, the leaves were rinsed with distilled water for three times and then sliced into small fragments (0.5 cm squares) and cultured on the MS medium supplemented with 1 mg/L NAA or 2,4-D  + 1 mg/L BAP or Kin and benomyl fungicide (100 or 500 mg/L).

Common disinfection method [including 70% (v/v) ethanol for 20 s  + 2% (w/v) NaOCl without pH adjustment for 12 min] was considered as control (Additional file 1: Table S2).

### Medium preparation and cultures incubation condition

MS medium were supplemented with 3% sucrose and solidified using 0.8% plant agar. The pH of the medium was adjusted to 5.7–5.8 before autoclaving at 121 °C for 20 min. Cultures were maintained in a growth chamber at 24 ± 1 °C and 16-h photoperiod (cool Wight florescent light; 55 μmol/m^2^s) and sub-cultured at monthly intervals. The percentage of callus induction, bacterial and fungal contaminations, viable and browning explants was recorded 4 weeks after culture. The callus fresh weight (mg/single explant) was measured 3 months after culture.

### Secondary metabolites analysis

According to the results of the experiments 1 and 2, the leaf explants were surface sterilized using A_2_ sterilization method, and cultured on the MS medium containing different combinations of plant growth regulators (PGRs) for callus induction and growth. The cultures were maintained in above maintained conditions and sub-cultured at 4 weeks interval. The secondary metabolites of the calli derived from leaf explants were extracted using Wagner (1979) method. Briefly, 0.1 g of fresh callus pulverized with liquid nitrogen, and suspended in 5 mL acidified methanol (methanol 99:1 acetic acid). The samples were incubated at room temperature for 72 h and centrifuged at 4000 rpm for 10 min. The supernatant was collected and used for determination of total phenolic, flavonoids and anthocyanin content.

Total phenolic content (TPC) were measured using colorimetric Folin-Ciocalteu spectrophotometric method described by Al-Farsi et al. (2005) and quantified using the standard curve of gallic acid (Sigma) (0, 0.001, 0.0015, 0.002 and 0.003 g/L of methanol) (y  = 14.333x − 0.00003 and R^2^  = 0.9933). Briefly, 3 mL of diluted Folin-Ciocalteu (1:10 with deionized water) were added to the 400 μL extract and incubated for 5 min in a water bath at 22 °C. Then, 3 mL sodium bicarbonate solution (7%) was added and the samples were incubated at 22 °C for 90 min. The absorbance of the samples were recorded at 725 nm using a spectrophotometer (SmartSpec Plus spectrophotometer, Bio-Rad, Hercules, CA, USA). Total phenolic content of the callus cells was expressed as mg of gallic acid equivalents (GAE) per 1 g fresh weight (FW). TPC calculated using equation 1 (Shubhangi et al. 2017):

1 $$ {\text{T}} = \left( {{\text{C}} \times {\text{V}}} \right)/{\text{M}} $$

where T is total phenolic content (mg GAE/g FW), C is the concentration of total phenolic content (TPC) (mg/mL), V is the final volume of the extract (mL), and M is the fresh weight of callus sample (g).

The total flavonoid content (TFC) in the callus cultures was determined using an aluminum chloride (AlCl_3_) colorimetric method reported by Anjum et al. (2017). Briefly, 0.25 mL of 10% AlCl_3_ and 0.25 mL of 1 M potassium acetate (CH_3_COOK) were added to 1 mL of methanolic extract. Then, the absorbance of the mixture was measured immediately at 498 nm against the control sample containing acidified methanol instead of the callus extract. The standard curve was prepared using different concentrations (0, 0.4, 0.8, 1.6, and 2.4 g/L) of quercetin in methanol and used for quantification of TFC in the callus samples (y  = 0.0573x  + 0.0025, R^2^  = 0.9888). The TFC of the callus expressed as mg of quercetin equivalents/1 g FW using the equation 1, where C is the concentration of flavonoid (mg/mL), V is the final volume of extract (mL), and M is the weight of the sample (g FW).

In order to quantification of anthocyanin content, the absorbance of the extracts measured at 550 nm. Then, the concentration of anthocyanin was calculated using the formula A  = ɛbc and molar extinction coefficient (ɛ_550_ = 33,000 × 10^6^ M^−1^ cm^−1^), and expressed as µM/g FW. Where A is the absorbance of the extracts, b is the width of the cuvette (cm), and c is the concentration of anthocyanin (M).

### HPLC analysis of rutin, quercetin and kaempferol

The separation, determination, and quantification of rutin, quercetin and kaempferol performed using high-performance liquid chromatography (HPLC) analysis. The secondary metabolites of the calli were extracted using Zu et al. (2006) method. Briefly, 0.5 g of the fresh callus pulverized in liquid nitrogen, and 1.5 mL ethanol (90% v/v) were added and vortexed for 5–10 min. Then, the samples were sonicated in a bath sonicator (Bandelin electronic^®^, Germany) for 15 min at 35 °C and incubated at room temperature for 3 h. The vortex and sonication were repeated twice. Finally, the samples were centrifuged (Sigma 1-14 K, Germany) at 10,000 rpm for 15 min. The supernatant was collected and filtered using a 0.22 μm filter. The filtrate was dried at 45 °C, till final volume was up to 100 μL and stored at − 20 °C until analysis. The standard curve was prepared using different concentrations of rutin (Sigma-Aldrich, USA) (5, 50, 100 ppm); quercetin (Sigma-Aldrich, Q4951, CAS: 117-39-5) and kaempferol (Sigma-Aldrich, 60010, CAS: 520-18-3) (5, 50, 100 and 500 ppm) and used for quantification in the callus samples.

HPLC analyses were performed on a Sykam HPLC system (SYKAM 1130, Sykam GmbH, Germany). HPLC separations carried out on a HIQ SIL C18V reversed-phase column (ø 4.6 × 250 mm) packed with 5 μm diameter particles, the mobile phase was methanol–acetonitrile–water (40:15:45, v/v/v). Rutin, quercetin and kaempferol were detected by diode-array detector DAD following HPLC separation at 257 nm for rutin and 368 nm for quercetin and kaempferol. Flow rate and injection volume was 1.0 mL/min and 20 μL, respectively. All chromatographic operations were carried out at room temperature.

### Statistical analysis

Disinfection experiments conducted in a factorial (disinfection and PGRs as factors) arrangement based on a completely randomized design with six replicates with 12 explants per replication. Secondary metabolites (TPC, TFC, AC, rutin, quercetin and kaempferol) measurements carried out in a completely randomized design (CRD) with three replications. Experimental data subjected to analysis of variance (ANOVA) (p  < 0.05) and mean comparison using Duncan’s Multiple Range Test (DMRT) (at p  < 0.05) using IBM SPSS Ver.21 statistical software (IBM Corporation and Others, Armonk, NY, USA). The results expressed as mean  ±  SE. The graphs produced using Microsoft Office Excel 2010.

## Results

### Disinfection

#### ***Experiment 1: the effects of different concentrations of H***_***2***_***O***_***2***_*** and NaOCl, and pH adjustment of NaOCl on the contamination and growth of explants***

Some of the cultures were contamination–free and the explants exhibited rapid swelling and cell proliferation especially from the edge of cuttings, which was led to the callus formation within 2–3 weeks (Additional file 1: Fig. S1). Embryogenic calli had green spots that showed signs of differentiation and morphogenesis after culture on the medium containing high proportion of cytokinin/auxin (Additional file 1: Fig. S2). The results indicated that the percentage of bacterial and fungal contamination, clean explants, explants viability and browning and callus induction and growth were significantly influenced by disinfection methods. Furthermore, there were significant differences between PGRs treatments in terms of explants viability and browning (%), and callus induction and growth (Additional file 1: Table S3). The highest bacterial (15.97%) and fungal (29.49%) contaminations occurred in the control sterilization method. The highest percentage of clean explants was achieved in the A_3_–A_7_ disinfection methods, while the highest percentage of viable explants was obtained in the control, A_1_, A_2_ and A_5_ disinfection methods (Table 1). Furthermore, the lowest percentage of explant browning and the highest percentage of callus induction and growth achieved with the A_2_ disinfection method (Table 1). As shown in Table 1, in the methods including NaOCl pH adjustment (A_3_, A_5_ and A_7_) the minimum percentage of explants with fungal and bacterial contamination was occurred, but the percentage of explants with callus induction and growth response was also lower. For instance, the application of 2% NaOCl with pH  = 7 for 12 min (method A_3_) significantly decreased the explant viability and increased the browning explants as compared to 2% NaOCl without pH adjustment (method A_2_). Furthermore, the methods A_2_ and A_3_ in which employed benomyl pretreatment and H_2_O_2_ had an inhibitory effect on the bacterial contamination compared to the method A_1_ in which only explants pretreated with benomyl solution (3 mg/L) for 2 h (Table 1). Furthermore, the highest percentage of callus induction was obtained in MS medium supplemented with 3 mg/L NAA  + 1 mg/L Kin or BAP and 3 mg/L NAA or 2,4-D  + 3 mg/L BAP (Table 2). While, the highest callus yield (fresh weight) was obtained in the MS medium containing 5 mg/L 2,4-D + 5 mg/L Kin and 5 mg/L NAA + 5 mg/L BAP (Table 2).

**Table 1:** The effect of different disinfection methods on the percentage of microbial contamination and in vitro response of *M. azedarach* L. leaf explants

Methods of disinfection	Bacterial contamination (%)	Fungal contamination (%)	Clean explant (%)	Viability (%)	Browning (%)	Callus induction (%)	weight of callus (mg/explant)
Control	15.97 ± 3.10^d^	29.49 ± 4.08^c^	54.5 ± 4.8^d^	1.00 ± 0.00^a^	0.00 ± 0.00^a^	26.78 ± 5.58^b^	873.733 ± 34.418^ab^
A_1_	8.74 ± 1.56^c^	1.08 ± 0.45^a^	90.2 ± 1.6^bc^	1.00 ± 0.00^a^	0.00 ± 0.00^a^	5.91 ± 1.59^c^	484.568 ± 26.896^bc^
A_2_	5.57 ± 1.21^bc^	7.45 ± 1.47^b^	87.0 ± 2.4^c^	99.65 ± 0.35^a^	0.35 ± 0.35^a^	38.56 ± 5.49^a^	1191.294 ± 37.148^a^
A_3_	3.39 ± 0.94^ab^	0.00 ± 0.00^a^	96.6 ± 0.9^ab^	86.45 ± 3.29^b^	14.20 ± 3.25^b^	1.52 ± 0.91^c^	158.921 ± 96.003^c^
A_4_	0.40 ± 0.40^a^	0.00 ± 0.00^a^	99.6 ± 0.4^a^	79.25 ± 3.58^c^	21.14 ± 3.67^c^	9.41 ± 2.37^c^	554.056 ± 89.829^bc^
A_5_	0.86 ± 0.60^a^	0.69 ± 0.48^a^	98.5 ± 0.8^a^	99.31 ± 0.48^a^	0.79 ± 0.55^a^	10.77 ± 2.82^c^	282.111 ± 100.367^c^
A_6_	1.06 ± 0.60^a^	1.32 ± 0.63^a^	97.6 ± 0.8^a^	77.87 ± 3.94^c^	22.72 ± 4.01^c^	3.91 ± 1.43^c^	130.382 ± 60.386^c^
A_7_	0.37 ± 0.37^a^	0.00 ± 0.00^a^	99.6 ± 0.4^a^	54.48 ± 4.64^d^	45.52 ± 4.64^d^	9.44 ± 3.76^c^	108.147 ± 47.128^c^

In each column, different letters indicate a significant difference (*P* ≤ 5%) according to Duncan’s Multiple-Range Tests (DMRTs)

**Table 2:** The effect of the different types and concentrations of PGRs on the explant viability, browning, and callus induction from leaf explants of *M. azedarach* L.

Concentration of PGRs (mg/L)	Viability (%)	Browning (%)	Callus induction (%)	Callus weight (mg/explant)
3 NAA + 1 Kin	91.33 ± 4.00^ab^	8.67 ± 4.00^cd^	18.17 ± 3.60^a^	455.737 ± 17.445^b−e^
3 NAA + 1 BAP	83.70 ± 5.98^bc^	16.30 ± 0.50^abc^	19.00 ± 3.79^a^	814.529 ± 252.15^b−e^
3 2,4-D + 1 Kin	88.30 ± 5.03^ab^	11.89 ± 3.18^bcd^	15.32 ± 3.89^ab^	66.444 ± 39.585^de^
3 2,4-D + 1 BAP	90.82 ± 3.50^ab^	9.17 ± 3.53^cd^	16.00 ± 3.83^ab^	103.150 ± 41.373^de^
3 NAA + 3 Kin	78.80 ± 6.07^c^	21.19 ± 4.07^a^	14.49 ± 3.92^abc^	987.000 ± 28.824^bc^
3 NAA + 3 BAP	77.66 ± 6.18^c^	22.33 ± 4.18^a^	18.35 ± 3.13^a^	851.095 ± 30.287^bcd^
3 2,4-D + 3 Kin	76.80 ± 5.23^c^	23.19 ± 3.43^a^	10.14 ± 3.46^abc^	78.800 ± 48.109^de^
3 2,4-D + 3 BAP	83.21 ± 5.62^bc^	18.06 ± 3.01^ab^	20.15 ± 3.14^a^	488.714 ± 19.267^b−e^
5 NAA + 1 Kin	91.97 ± 4.18^ab^	9.03 ± 4.18^cd^	11.85 ± 3.69^abc^	154.706 ± 83.611^de^
5 NAA + 1 BAP	89.30 ± 4.07^ab^	10.70 ± 4.07^bcd^	3.67 ± 3.67^bc^	53.647 ± 53.647^de^
5 2,4-D + 1 Kin	91.66 ± 3.76^ab^	8.33 ± 3.76^cd^	0.98 ± 0.98^c^	2.765 ± 2.765^e^
5 2,4-D + 1 BAP	91.24 ± 4.04^ab^	9.93 ± 4.48^bcd^	10.29 ± 3.31^abc^	112.353 ± 53.227^de^
5 NAA + 5 Kin	96.98 ± 1.65^a^	3.32 ± 1.58^d^	15.47 ± 3.68^ab^	222.167 ± 92.369^cde^
5 NAA + 5 BAP	96.14 ± 1.84^a^	5.25 ± 1.91^d^	14.09 ± 3.41^abc^	1073.722 ± 63.623^b^
5 2,4-D + 5 Kin	91.20 ± 5.05^ab^	8.79 ± 3.05^cd^	15.98 ± 3.46^ab^	1804.833 ± 85.304^a^
5 2,4-D + 5 BAP	92.90 ± 3.14^a^	7.09 ± 3.14^d^	11.73 ± 2.90^abc^	342.063 ± 179.899^b−e^

In each column, different letters indicate a significant difference (*P* ≤ 5%) according to Duncan’s Multiple-Range Tests (DMRTs)

#### Experiment 2: the effect of benomyl inclusion in the culture medium on the contamination and growth of explants

**Fig. 1:**
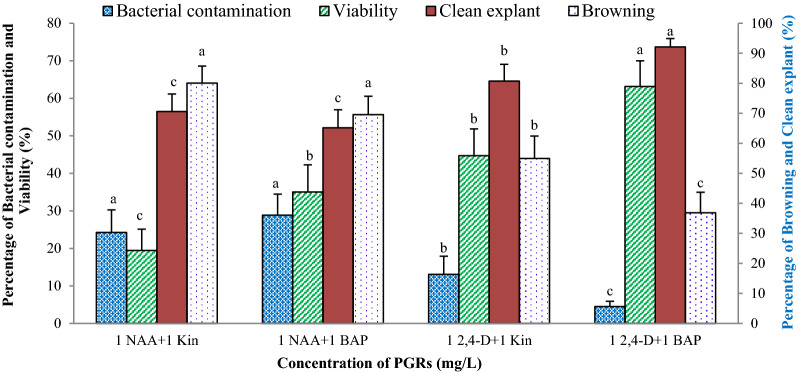
The effect of plant growth regulators on in vitro responses of *M. azedarach* L. leaf explants in the culture media containing benomyl (Experiment 2). Bars with different letters indicate a significant difference (*P* ≤ 5%) according to Duncan’s Multiple-Range Tests (DMRTs)

**Fig. 2:**
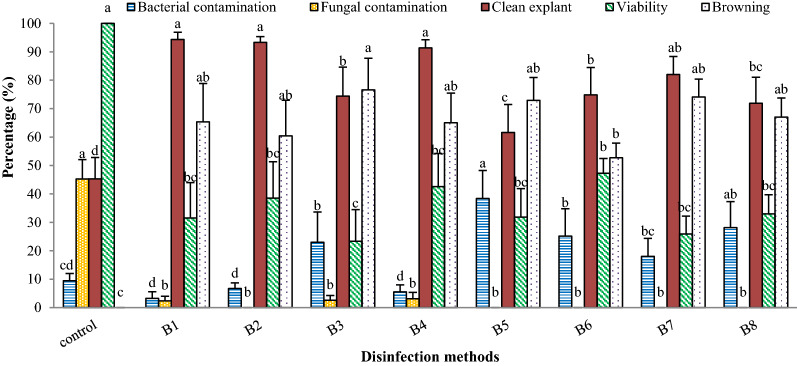
The effect of different disinfection methods and inclusion of benomyl in culture medium on the bacterial contamination and in vitro response of *M. azedarach* L. leaf explants. Bars with different letters indicate a significant difference (*P* ≤ 5%) according to Duncan’s Multiple-Range Tests (DMRTs)

In this experiment, the effect of disinfection methods and the inclusion of benomyl in the culture medium on the explant contamination and viability were assessed. The percentage of bacterial contamination, and viable and browning explants were significantly influenced by the disinfection method, PGRs combination, and their interaction. The percentage of explant browning in the medium containing NAA was higher than that of the medium supplemented with 2,4-D, while the explant viability in the media containing 2,4-D was higher than those containing NAA. Furthermore, explant viability influenced by cytokinin type, so in both 2,4-D and NAA containing media, BAP improved the explant viability as compared to kinetin (Fig. 1). As a result, the maximum and minimum explant viability was observed in the medium supplemented with 1 mg/L 2,4-D  + 1 mg/L BAP and 1 mg/L NAA  + 1 mg/L Kin, respectively. Inclusion of benomyl (100 and 500 mg/L) in the culture medium composition significantly reduced the percentage of fungal contamination in *M. azedarach* L. leaf explant cultures. So, the percentage of viable explants and fungal contamination in the control method were higher than the others. Disinfection methods B_1_ and B_5_ showed the lowest and highest bacterial contamination (%), respectively (Fig. 2). In this experiment, the callus induction only obtained from the explants disinfected with the method B_4_ and cultured on the MS medium supplemented with 1 mg/L NAA  + 1 mg/L BAP and 1 mg/L 2,4-D  + 1 mg/L Kin or BAP.

#### Total phenolic content (TPC), total flavonoid content (TFC) and Anthocyanin (AC)

Spectrophotometric assay of the secondary metabolites content in the *M. azedarach* L. calli obtained from different PGRs combinations (Additional file 1: Fig. S3) indicated that the amount of TPC, TFC, and AC were significantly influenced by the PGRs (Additional file 1: Table S4). According to the results, TPC in the methanolic extract was varied from 0.001 to 0.109 mg GAE/g FW in 5 mg/L NAA  + 5 mg/L Kin and 3 mg/L 2,4-D  + 3 mg/L BAP treatments, respectively. The highest amount of TFC was obtained in 3 mg/L 2,4-D  + 3 mg/L BAP (193.051 mg QE/g FW) treatment, while the lowest value was observed in 5 mg/L NAA  + 1 mg/L Kin and 5 mg/L 2,4-D  + 5 mg/L Kin (58.583 and 64.638 mg QE/g FW, respectively) treatments. The highest and lowest amount of anthocyanin (AC), were obtained from the calli grown on the MS medium supplemented with 3 mg/L 2,4-D  + 1 mg/L BAP or 3 mg/L Kin and 3 mg/L NAA  + 3 mg/L BAP, respectively (Table 3).

**Table 3:** The effect of plant growth regulators on total phenolic (TPC), Total flavonoid (TFC), and anthocyanin (AC) content in the callus cultures of *M. azedarach* L.

Concentration of PGRs (mg/L)	TPC (mg GAE/g FW)	TFC (mg QE/g FW)	AC (μm/g FW)
1 NAA + 1 KIN	0.010 ± 0.005^fg^	82.805 ± 4.545^b−g^	1.650 ± 0.170^bcd^
1 NAA + 1 BAP	0.021 ± 0.006^d−h^	80.402 ± 11.385^b−g^	1.508 ± 0.302^bcd^
1 2,4-D + 1 KIN	0.027 ± 0.009^d−g^	67.522 ± 4.774^efg^	1.647 ± 0.042^bcd^
1 2,4-D + 1 BAP	0.030 ± 0.006^d−g^	68.868 ± 5.132^d−g^	1.111 ± 0.208^cd^
3 NAA + 1 KIN	0.026 ± 0.007^d−g^	66.465 ± 10.599^fg^	1.084 ± 0.116^cd^
3 NAA + 1 BAP	0.051 ± 0.002^cd^	102.509 ± 13.155^b−e^	1.623 ± 0.563^bcd^
3 NAA + 3 KIN	0.066 ± 0.011^bc^	101.644 ± 17.790^b−g^	1.946 ± 0.481^abc^
3 NAA + 3 BAP	0.022 ± 0.010^d−g^	101.836 ± 14.609^b−e^	0.152 ± 0.077^e^
3 2,4-D + 1 KIN	0.094 ± 0.007^ab^	107.507 ± 9.145^bc^	2.458 ± 0.281^ab^
3 2,4-D + 1 BAP	0.043 ± 0.002^c−f^	100.202 ± 2.888^b−f^	2.707 ± 0.386^a^
3 2,4-D + 3 KIN	0.090 ± 0.001^ab^	109.237 ± 6.528^b^	2.667 ± 0.545^a^
3 2,4-D + 3 BAP	0.109 ± 0.007^a^	193.051 ± 16.732^a^	2.455 ± 0.293^ab^
5 NAA + 1 KIN	0.013 ± 0.007^efg^	58.583 ± 13.115^g^	1.525 ± 0.417^bcd^
5 NAA + 1 BAP	0.012 ± 0.006^fg^	68.291 ± 9.497^d−g^	1.6061 ± 0.102^bcd^
5 NAA + 5 KIN	0.001 ± 0.014^g^	73.385 ± 13.732^c−g^	1.448 ± 0.288^cd^
5 NAA + 5 BAP	0.017 ± 0.012^efg^	69.829 ± 10.656^d−g^	1.216 ± 0.218^cd^
5 2,4-D + 1 KIN	0.046 ± 0.009^cde^	100.875 ± 2.217^b−f^	1.865 ± 0.084^abc^
5 2,4-D + 1 BAP	0.041 ± 0.016^c−f^	103.278 ± 0.254^bcd^	1.872 ± 0.148^abc^
5 2,4-D + 5 KIN	0.022 ± 0.019^d−g^	64.639 ± 11.731^g^	0.673 ± 0.139^de^
5 2,4-D + 5 BAP	0.017 ± 0.008^efg^	70.406 ± 2.141^d−g^	0.741 ± 0.255^de^

In each column, different letters indicate a significant difference (*P* ≤ 5%) according to Duncan’s Multiple-Range Tests (DMRTs)

As shown in Table 3, increasing the concentration of 2,4-D from 1 to 3 mg/L significantly increased the amount of TPC, TFC, and AC contents. After that, increasing the 2,4-D concentration from 3 to 5 mg/L significantly decreased the production and accumulation of these secondary metabolites in the callus cultures of *M. azedarach* L. (Table 3). However, these increase and decreases were varied depending on the type and concentration of cytokinin. For instance, increasing the concentration of 2,4-D from 3 to 5 mg/L in the medium containing 5 mg/L Kin or BAP significantly decreased the AC content, but this reduction was not statistically significant in the medium containing 1 mg/L Kin or BAP (Table 3). On the other hand, increasing the 2,4-D concentration from 3 to 5 mg/L significantly reduced the TPC and AC contents of the callus cells in both media containing 1 and 5 mg/L of Kin or BAP. Furthermore, the amount of TFC in the callus cells grown on the medium containing 5 mg/L 2,4-D  + 5 mg/L Kin or BAP was significantly lower than that of containing 5 mg/L 2,4-D  + 1 mg/L Kin or BAP. While, there were no significant differences between 1, 3, and 5 mg/L concentrations of Kin or BAP in the media containing 5 mg/L NAA (Table 3).

#### Rutin, quercetin and kaempferol content

**Fig. 3:**
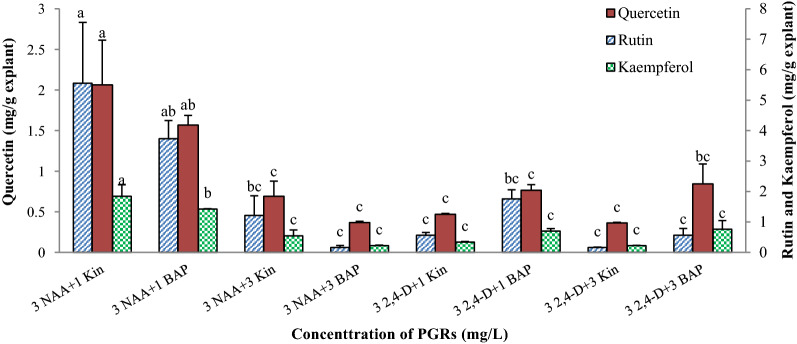
The effect of the plant growth regulators type and concentrations on the amount of rutin, quercetin and kaempferol in the callus cultures of *M. azedarach* L. Bars with different letters indicate a significant difference (*P* ≤ 5%) according to Duncan’s Multiple-Range Tests (DMRTs)

The amount of rutin, quercetin and kaempferol in callus cultures were analyzed using HPLC (Additional file 1: Fig. S4a, S4b), and the results indicated that the production and accumulation of these secondary metabolites significantly influenced by both type and concentration of plant growth regulators (Additional file 1: Table S5). So that, the highest amount of rutin, quercetin and kaempferol were obtained from the medium supplemented with 3 mg/L NAA  + 1 mg/L Kin and 3 mg/L NAA  + 1 mg/L BAP treatments (Fig. 3).

## Discussion

**Fig. 4:**
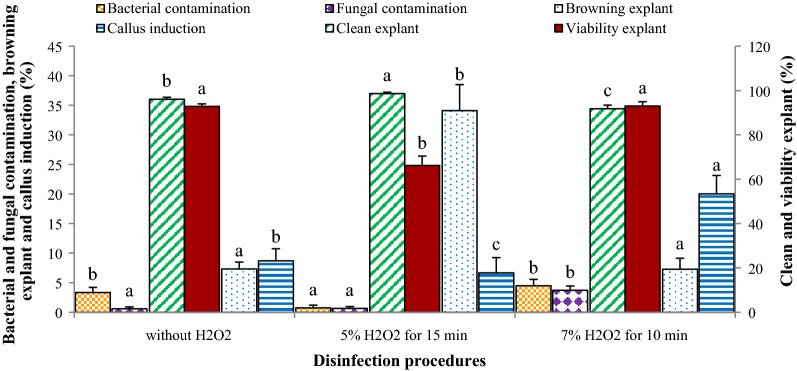
The effect of H_2_O_2_ inclusion and concentration in disinfection protocol on the *M. azedarach* L. leaf explants contamination and viability. Bars with different letters indicate a significant difference (*P* ≤ 5%) according to Duncan’s Multiple-Range Tests (DMRTs)

Tissue culture techniques play a vital role in plant biotechnology, especially in medicinal plants. Therefore, establishment of successful cultures and overcome to in vitro contamination requires an efficient protocol for explant disinfection. On the other hand, the preparation and establishment of clean in vitro cultures, especially in woody plants and plant materials prepared from natural habitats is a critical stage in successful plant cell and tissue culture. Disinfection procedures may differ for different plants, depending on their morpho-physiological characteristics (Srivastava et al. 2010). H_2_O_2_ has been reported in a few reports as a potent oxidizing and sterilization agent for the surface disinfection of different plant materials (Mihaljević et al. 2013; Hesami et al. 2019). In our study, utilization of H_2_O_2_ (A_2_, A_3_, A_6_, and A_7_ methods) increased the percentage of browning explants (from 7.312 to 20.698%) and decreased the explants viability and bacterial contamination (from 92.854 to 79.614% and 3.336 to 2.599%, respectively) in comparison to the methods without this agent (A_1_, A_4_, and A_5_ methods) (Table 1; Fig. 4). Increasing the concentration of H_2_O_2_ from 5% (methods A_6_ and A_7_) to 7% (method A_2_ and A_3_) significantly decreased the percentage of browning explants (from 34.121 to 7.275%) and subsequently increased the percentage of explants viability and callus induction (Table 1; Fig. 4). Curvetto et al. (2006) reported that clean bulblet explants in *Lilium* improved with increasing the H_2_O_2_ concentration. Accordingly, in Bakhsh et al. (2016) study, the highest contamination-free of *gossypium hirsutum* L. explants were achieved by utilization of H_2_O_2_. In our study, *M. azedarach* L. leaf explants viability and callus induction in methods including 7% H_2_O_2_ for 10 min were 1.5-times and 3-times, respectively, higher than those of 5% H_2_O_2_ for 15 min. On the other hand, a higher concentration of H_2_O_2_ with lower exposure duration has no adverse effects on explant viability and callus induction from *M. azedarach* L. leaf explants. Our results are in agreement with Hesami et al. (2019) and Mihaljević et al. (2013) observations in surface sterilization of chrysanthemum and sour cherry, respectively.

**Fig. 5:**
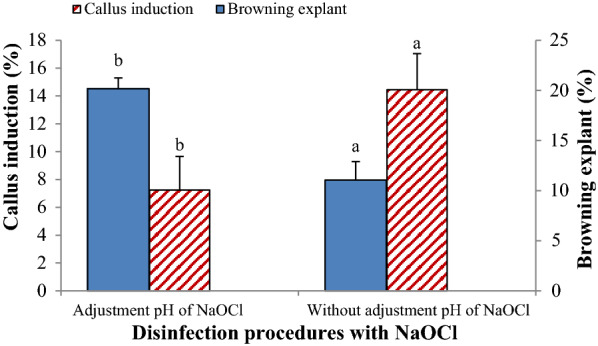
The effect of NaOCl pH adjustment on *M. azedarach* L. leaf explant disinfection and in vitro culture responses in the media containing benomyl. Bars with different letters indicate a significant difference (*P* ≤ 5%) according to Duncan’s Multiple-Range Tests (DMRTs)

In the present study utilization of a two-step disinfection procedure improved the disinfection efficiency and in vitro response of *M. azedarach* L. leaf explants. So that, the clean explants achieved from two agents surface disinfection [pretreatment (step one) with 0.5% NaOCl  + 3 mg/L benomyl and treatment (step 2) with 2% NaOCl for 12 min (methods A_4_ and A_5_)] was higher than those obtained from one step surface disinfection, in which only 2% NaOCl treatment was applied for 12 min (control treatment) (Table 1). Similarly, in Kozak and Stelmaszczuk (2013) study, 60% and 80% clean cultures of *Allium karataviense* explants were obtained, when 4 and 2% NaOCl applied for 30 min followed by 1% NaOCl for 15 min, respectively. Furthermore, Sivanesan et al. (2014) reported 100% clean cultures of *Crocus vernus* L. corms by utilization of 2% NaOCl for 10 min and 0.01% HgCl_2_. Similarly, Siavash Moghaddam et al. (2011) achieved 40% clean explant with utilization of 1 g/L benomyl and 10–15% clorox^®^, and this value enhanced up to 60% with the addition of plant preservative mixture (PPM) to the culture medium. Furthermore, in the present study, although adjustment of NaOCl pH to pH  = 7 and 10 significantly reduced the microbial contamination and increased the percentage of clean explants in *M. azedarach* L. in vitro cultures, but adversely influenced the explant viability and callus induction (Table 1; Fig. 5). It has been shown that with reducing the pH of NaOCl solution, the chlorine as ^–^OCl increases, which has been considered as a strong oxidizing agent and most effective on disinfecting functions (Fukuzaki 2006). It seems that these elevated levels of ^–^OCl in NaOCl solution with pH  = 7 and 10 exert cytotoxic effects on *M. azedarach* L. leaf explants; and led to the cell death and reduced in vitro responses of the explants.

Fungicides and bactericides can be useful to proper disinfection and have been used in previous investigations (Sharan et al. 2010). In the aqueous solutions, benomyl breaks down into two compounds, methyle-2-benzimidazolecarbamate and butyl isocyanate, which are toxic to fungi (Pence 2005). The results of the present study showed that, inclusion of benomyl in the culture medium significantly reduced the fungal contamination as compared the control treatment. So, about 80% clean explant was obtained by addition of 100 or 500 mg/L benomyl in the culture medium. Our findings are in agreement with Haldeman et al. (1987) observation, who reported 100% clean cultures of *Camellia sinensis* shoot tip explants in MS medium supplemented with 1 g/L benomyl. Furthermore, Siavash Moghaddam et al. (2011) achieved up to 50% clean explant in *Centella asiatica* L. by inclusion 100 mg/L benomyl in the culture medium composition.

Plants show different in vitro growth response to different types and concentrations of plant growth regulators, especially the combination of auxins and cytokinins (Guo et al. 2009; Firoozi et al. 2019). In the present study, all used PGRs treatment induced callogenesis from *M. azedarach* L. leaf explants, but with different percentage and callus growth rate. The moderate concentration of NAA or 2,4-D (3 mg/L) combined with 1 or 3 mg/L Kin/BAP resulted in the highest callus induction and growth. So that, increasing the concentration of NAA or 2,4-D up to 5 mg/L was led to reduced callus induction and growth and the lowest percentage of callus induction obtained in the higher ratios of Aux/Cyt (5 mg/L 2,4-D  + 1 mg/L Kin and 5 mg/L NAA  + 1 mg/L BAP). These results are in agreement with Firoozi et al. (2019) report in saffron. Nevertheless, Castellar and Iborra (1997) reported the highest callus induction from saffron in vitro cultures on the MS medium supplemented with 10 mg/L NAA and 5 mg/L BAP.

**Fig. 6:**
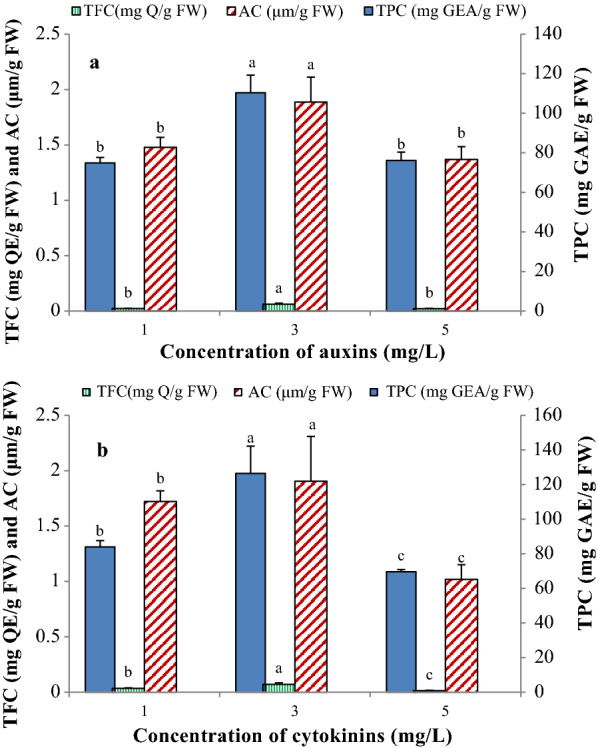
The effect of auxins (**A**) and cytokinins (**B**) concentration on TPC, TFC and AC content in the callus cultures of *M. azedarach* L. Bars with different letters indicate a significant difference (*P* ≤ 5%) according to Duncan’s Multiple-Range Tests (DMRTs)

Among the phytochemicals, flavonoids and phenolic compounds are well-known compounds which have antioxidant and anticancer properties may be due to their strong free radical scavenging activity (Tungmunnithum et al. 2018). There has been a growing interest in the improving production of these valuable phytochemicals in the plant cells through in vitro cell culture techniques. Manipulation and optimization of cell culture conditions and medium compositions such as PGRs provide a practical approach for manipulation and improving the cell growth and secondary metabolite production. PGRs act as one of the critical factors influencing the growth and development of plant cells in both whole plant and in vitro cultures (Farjaminezhad et al. 2013; Firoozi et al. 2019). Our results indicated that in vitro production of bioactive secondary metabolites in *M. azedarach* L. callus cultures was significantly influenced by the type and concentrations of auxins and cytokinins. So, the medium supplemented with moderate concentration (3 mg/L) of 2,4-D in combination with 1 or 3 mg/L BAP/Kin increased the amount of TPC, TFC, and AC in the callus cultures. So that, the amount of TFC and AC obtained from the calli grown on the medium supplemented with a moderate concentration of 2,4-D was 1.5–2 times higher than those attained from the medium supplemented with other concentrations of 2,4-D or NAA. Regardless of the type of auxins, increasing the auxin concentrations from 1 to 3 were enhanced the amount of TPC, TFC, and AC. In contrast, increasing the auxin concentration from 3 to 5 significantly reduced the TPC, TFC and AC production and accumulation in the *M. azedarach* L. callus cultures (Table 3; Fig. 6A). These results revealed that both type and concentration of auxin play a significant role in the production and accumulation of secondary metabolites in the *M. azedarach* L. callus cultures. Similarly, Javed et al. (2017) reported that the highest amount of TPC and TFC in *Stevia rebaudiana* in vitro cultures produced in 2 mg/L NAA + 0.5 mg/L BAP treatment. Where, the lowest TPC was obtained from 2 mg/L 2,4-D + 1 mg/L BAP, and 0.5 mg/L 2,4-D + 0.5 mg/L Kin. Also, the amount of TPC, TFC and AC were increased by increasing the cytokinin concentration from 1 to 3. In contrast, the amount of these metabolites declined by increasing the cytokinin concentration from 3 to 5 mg/L (Fig. 6B). Contrariwise, among the treatments assessed in the present study, the calli grown on MS medium containing 3 mg/L NAA  + 1 mg/L Kin produced the highest amount of rutin, quercetin and kaempferol (5.556, 2.063 and 1.843 mg/g FW, respectively), which respectively was about 34, 5.68 and 8.23 times higher than those obtained from 3 mg/L 2,4-D  + 3 mg/L Kin. Furthermore, the calli grown on MS medium supplemented with 3 mg/L NAA  + 1 mg/L BAP produced 3.733, 1.568 and 1.423 mg/g FW of rutin, quercetin and kaempferol, respectively. On the other hand, the production of these secondary metabolites inhibited through 2,4-D. The highest and lowest suppression was occurred in the MS medium containing 3 mg/L 2,4-D  + 3 mg/L Kin and 3 mg/L 2,4-D  + 1 mg/L BAP, respectively. Nair et al. (1992) suggested that the suppression effects of 2,4-D to secondary metabolite production may be attributed to the herbicidal properties of 2,4-D. Furthermore, the results of the present study revealed that with increasing the concentration of cytokinin from 1 to 3 mg/L, the amount of rutin (4.5-times), quercetin (4-times) and kaempferol (3.4-times) in callus cultures were decreased significantly (p ≤ 5%). These decreases were varied depending on the auxin type, so that, the amount of decline of rutin, quercetin and kaempferol in the treatments containing NAA were significantly higher than those of the containing 2,4-D. On the other hands, the calli grown on the medium containing Kin produced the higher amount of rutin, quercetin and kaempferol as compared to those on the medium containing BAP, especially at 1 mg/L level. Farjaminezhad and Garoosi (2021) reported the higher azadirachtin production in *Azadirachta indica* cell suspension culture in the medium containing 2 mg/L Kin. In Lian et al. (2002) study on *Panax ging*, saponin productivity increased when the medium supplemented with a low concentration of cytokinins, but the growth of the cells was not affected. Cytokinins have also been reported to be effective in the induction of artemisinin production in *Artemisia missouriensis *in vitro cultures (Zia et al. 2007). Zia et al. (2007) reported that inclusion of 8.88 µM BAP produced 3.05 µg/g artemisinin in *A. absinthium* callus cultures, which was higher than that of the media containing NAA or Kin.

In the present study, we established an efficient protocol for disinfection and in vitro culture of *M. azedarach* L. Utilization of two different disinfection agents (first with H_2_O_2_ fallowed by NaOCl) improved the sterilization efficiency and in vitro response in *M. azedarach* L. leaf explants. Here we demonstrated the production and accumulation of secondary metabolites including rutin, quercetin and kaempferol in callus cultures of *M. azedarach* L. Furthermore, the amount of these secondary metabolites influenced by both type and concentration of plant growth regulators. These findings provide a newfound knowledge and the practical approach for biotechnological studies in this medicinal plant and production of valuable secondary metabolites.

## Supplementary Information

**Additional file 1: Figure S1.** In vitro culture of *M. azedarach *L.; A_1_: leaf explants, B_1_: viable and browning leaf explants after disinfection and in vitro culture, C_1_: swollen leaf explants and initiation of callus induction on MS medium containing 1 mg/L NAA  + 1 mg/L Kin, D_1_ and E_1_: callus induced on MS medium containing 1 mg/L NAA  + 1 mg/L BAP and 3 mg/L NAA  + 1 mg/L BAP (is embryogenic callus), respectively, A_2_ and B_2_: callus initiation and growth on MS  + 1 mg/L 2,4-D  + 1 mg/L Kin, C_2_, D_2_ and E_2_: callus growth on MS medium containing 1 mg/L 2,4-D  + 1 mg/L Kin, 1 mg/L NAA  + 1 mg/L Kin and 3 mg/L 2,4-D  + 3 mg/L BAP, respectively. **Figure S2.** In vitro culture of *M. azedarach *L.; A_1_: callus induction on MS medium containing 3 mg/L NAA  + 1 mg/L Kin containing green cells in callus and B_1_: 3 mg/L NAA  + 1 mg/L BAP containing green and globular shape spots in callus (are embryogenic callus), C_1_: embryogenic callus that have taken root, A_2_: globular and torpedo shape embryos, B_2_: globular embryos under a stereoscope and C_2_: torpedo shape embryos under a stereoscope. **Figure S3. **In vitro culture of *M. azedarach *L.; Callus growth on MS  + A_1_: 1 mg/L NAA  + 1 mg/L Kin, B_1_: 1 mg/L NAA  + 1 mg/L BAP, C_1_: 1 mg/L 2,4-D  + 1 mg/L Kin, D_1_: 1 mg/L 2,4-D  + 1 mg/L BAP, A_2_: 3 mg/L NAA  + 1 mg/L Kin, B_2_: 3 mg/L NAA  + 1 mg/L BAP, C_2_: 3 mg/L NAA  + 3 mg/L Kin, D_2_: 3 mg/L NAA  + 3 mg/L BAP, A_3_: 3 mg/L 2,4-D  + 1 mg/L Kin, B_3_: 3 mg/L 2,4-D  + 1 mg/L BAP, C_3_: 3 mg/L 2,4-D  + 3 mg/L Kin, D_3_: 3 mg/L 2,4-D  + 3 mg/L BAP, A_4_: 5 mg/L NAA  + 1 mg/L Kin, B_4_: 5 mg/L NAA  + 1 mg/L BAP, C_4_: 5 mg/L NAA  + 5 mg/L Kin, D_4_: 5 mg/L NAA  + 5 mg/L BAP, A_5_: 5 mg/L 2,4-D  + 1 mg/L Kin, B_5_: 5 mg/L 2,4-D  + 1 mg/L BAP, C_5_: 5 mg/L 2,4-D  + 5 mg/L Kin, D_5_: 5 mg/L 2,4-D  + 5 mg/L BAP, respectively. **Figure S4.** HPLC chromatogram of rutin, quercetin and kaempferol in the standard mixture (**A**) and *M. azedarach* L. calli grown on the MS medium containing 3 mg/L NAA  + 3 mg/L Kin (**B**). **Table S1.** Different disinfection methods used in experiment 1 for sterilization of *M. azedarach* L. leaf explants. **Table S2.** Different disinfection methods and inclusion of benomyl in culture medium used in experiment 2 for sterilization of *M. azedarach* L. leaf explants. **Table S3.** The effect of different disinfection methods on disinfection indices of leaf explants in *M. azedarach* L. (Experiment 1). **Table S4.** Effect of PGRs treatment on total flavonoid, total phenol and anthocyanin contents in *M. azedarach* L. calli. **Table S5.** Effect of PGRs treatment on rutin, quercetin and kaempferol in *M. azedarach* L. by HPLC analysis.

## Data Availability

All data generated or analyzed during this study are included in this published article.
