# Supplementary material for: Sterilization protocols and the effect of plant growth regulators on callus induction and secondary metabolites production in in vitro cultures Melia azedarach L
Source: AMB Express. 2022 Jan 10;12:3. doi: 10.1186/s13568-022-01343-8 (PMC8748552; doi:10.1186/s13568-022-01343-8)
Supplement: Supplementary file 1 — Additional file 1: Figure S1. In vitro culture of M. azedarach L.; A1: leaf explants, B1: viable and browning leaf explants after disinfection and in vitro culture, C1: swollen leaf explants and initiation of callus induction on MS medium containing 1 mg/L NAA + 1 mg/L Kin, D1 and E1: callus induced on MS medium containing 1 mg/L NAA + 1 mg/L BAP and 3 mg/L NAA + 1 mg/L BAP (is embryogenic callus), respectively, A2 and B2: callus initiation and growth on MS + 1 mg/L 2,4-D + 1 mg/L Kin, C2, D2 and E2: callus growth on MS medium containing 1 mg/L 2,4-D + 1 mg/L Kin, 1 mg/L NAA + 1 mg/L Kin and 3 mg/L 2,4-D + 3 mg/L BAP, respectively. Figure S2. In vitro culture of M. azedarach L.; A1: callus induction on MS medium containing 3 mg/L NAA + 1 mg/L Kin containing green cells in callus and B1: 3 mg/L NAA + 1 mg/L BAP containing green and globular shape spots in callus (are embryogenic callus), C1: embryogenic callus that have taken root, A2: globular and torpedo shape embryos, B2: globular embryos under a stereoscope and C2: torpedo shape embryos under a stereoscope. Figure S3. In vitro culture of M. azedarach L.; Callus growth on MS + A1: 1 mg/L NAA + 1 mg/L Kin, B1: 1 mg/L NAA + 1 mg/L BAP, C1: 1 mg/L 2,4-D + 1 mg/L Kin, D1: 1 mg/L 2,4-D + 1 mg/L BAP, A2: 3 mg/L NAA + 1 mg/L Kin, B2: 3 mg/L NAA + 1 mg/L BAP, C2: 3 mg/L NAA + 3 mg/L Kin, D2: 3 mg/L NAA + 3 mg/L BAP, A3: 3 mg/L 2,4-D + 1 mg/L Kin, B3: 3 mg/L 2,4-D + 1 mg/L BAP, C3: 3 mg/L 2,4-D + 3 mg/L Kin, D3: 3 mg/L 2,4-D + 3 mg/L BAP, A4: 5 mg/L NAA + 1 mg/L Kin, B4: 5 mg/L NAA + 1 mg/L BAP, C4: 5 mg/L NAA + 5 mg/L Kin, D4: 5 mg/L NAA + 5 mg/L BAP, A5: 5 mg/L 2,4-D + 1 mg/L Kin, B5: 5 mg/L 2,4-D + 1 mg/L BAP, C5: 5 mg/L 2,4-D + 5 mg/L Kin, D5: 5 mg/L 2,4-D + 5 mg/L BAP, respectively. Figure S4. HPLC chromatogram of rutin, quercetin and kaempferol in the standard mixture (A) and M. azedarach L. calli grown on the MS medium containing 3 mg/L NAA + 3 mg/L Kin (B). Table S1. Different disinfection methods used in experimen [file 13568_2022_1343_MOESM1_ESM.docx]

**AMB Express**

**Supporting Information:**

**Sterilization protocols and the effect of plant growth regulators on callus induction and secondary metabolites production in *in vitro* cultures of *Melia azedarach* L.**

**Fatemeh Ahmadpoor, Nasser Zare^*^, Rasool Asghari, Parisa Sheikhzadeh**

Department of Plant Production and Genetics, Faculty of Agriculture and Natural Resources, University of Mohaghegh Ardabili, P.O. Box 179, Ardabil, Iran

***Corresponding author:**

Dr. Nasser Zare

Associate Professor

Department of Plant Production and Genetics, Faculty of Agriculture and Natural Resources, University of Mohaghegh Ardabili, P.O. Box 179, Ardabil, Iran

E-mail: [zarenasser@yahoo.com](mailto:zarenasser@yahoo.com); [nzare@uma.ac.ir](mailto:nzare@uma.ac.ir)

Tel: +98(45) 31505113, Fax: +98(45)33512204


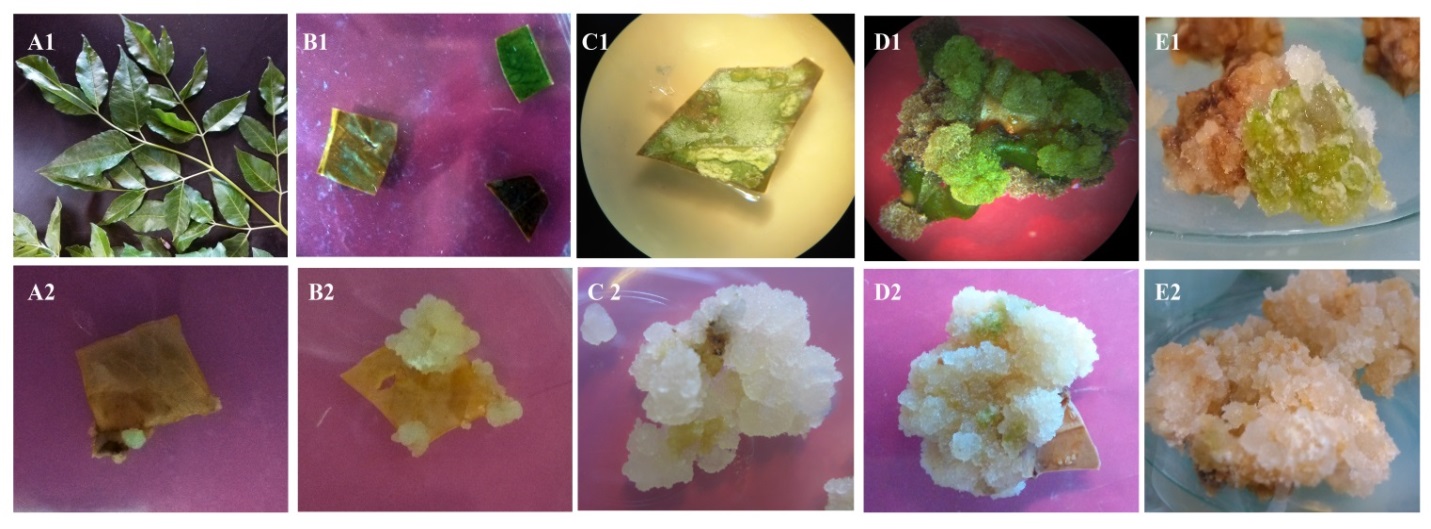


**Figure S1-** *In vitro* culture of *M. azedarach* L.*;* A_1_: Leaf explants, B_1_: Viable and browning leaf explants after disinfection and *in vitro* culture, C_1_: Swollen leaf explants and initiation of callus induction on MS medium containing 1 mg/L NAA+1 mg/L Kin, D_1_ and E_1_: Callus induced on MS medium containing 1mg/L NAA+1 mg/L BAP and 3 mg/L NAA+1 mg/L BAP (is embryogenic callus), respectively, A_2_ and B_2_: Callus initiation and growth on MS+1 mg/L 2,4-D+1 mg/L Kin, C_2_, D_2_ and E_2_: Callus growth on MS medium containing 1 mg/L 2,4-D + 1 mg/L Kin, 1 mg/L NAA+1 mg/L Kin and 3 mg/L 2,4-D+3 mg/L BAP, respectively.


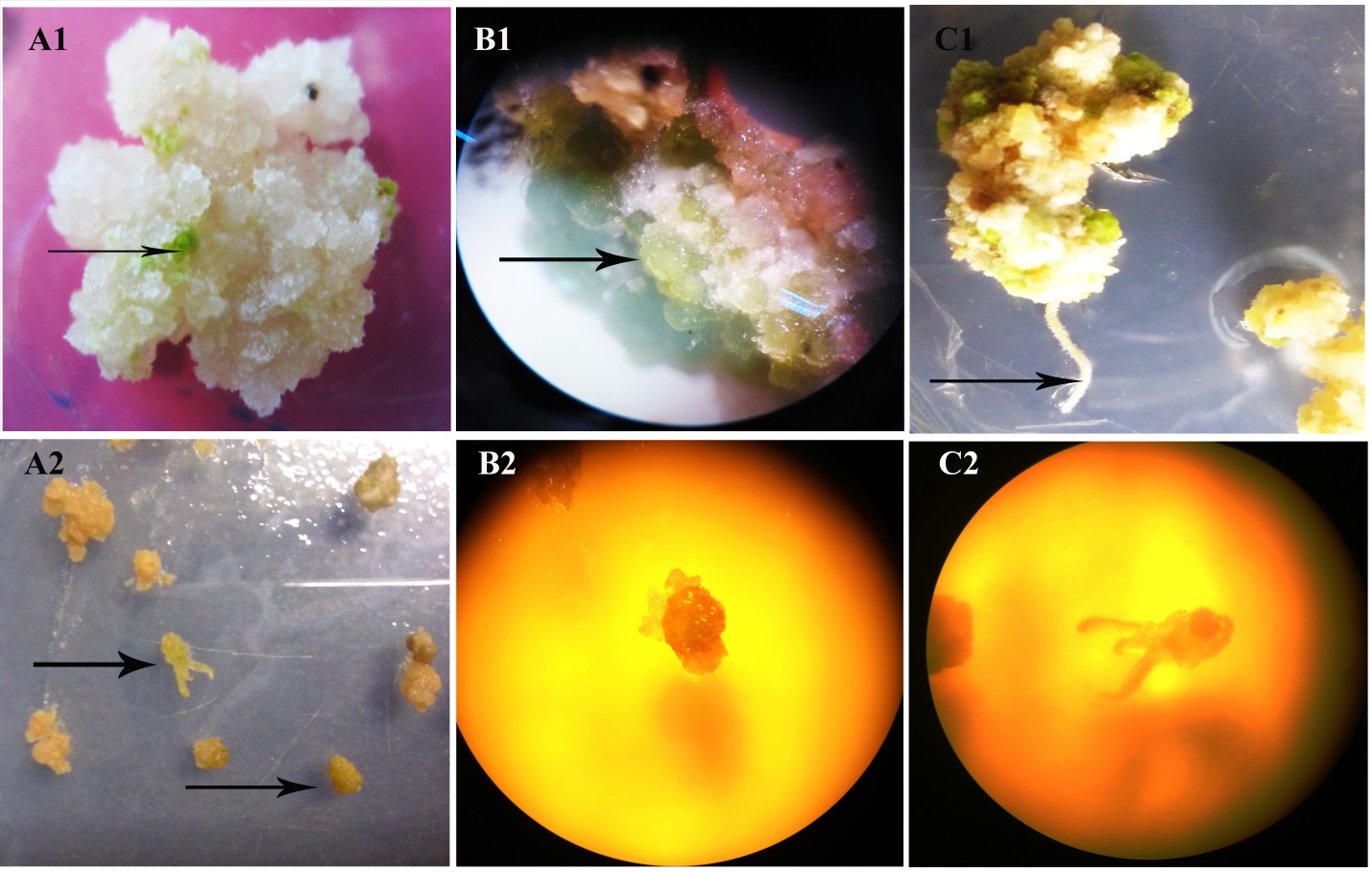


**Figure S2-** *In vitro* culture of *M. azedarach* L.*;* A_1_: Callus induction on MS medium containing 3 mg/L NAA+1 mg/L Kin containing green cells in callus and B_1_: 3 mg/L NAA+1 mg/L BAP containing green and globular shape spots in callus (are embryogenic callus), C_1_: Embryogenic callus that have taken root, A_2_:  Globular and torpedo shape embryos, B_2_: Globular embryos under a stereoscope and C_2_: torpedo shape embryos under a stereoscope.

**
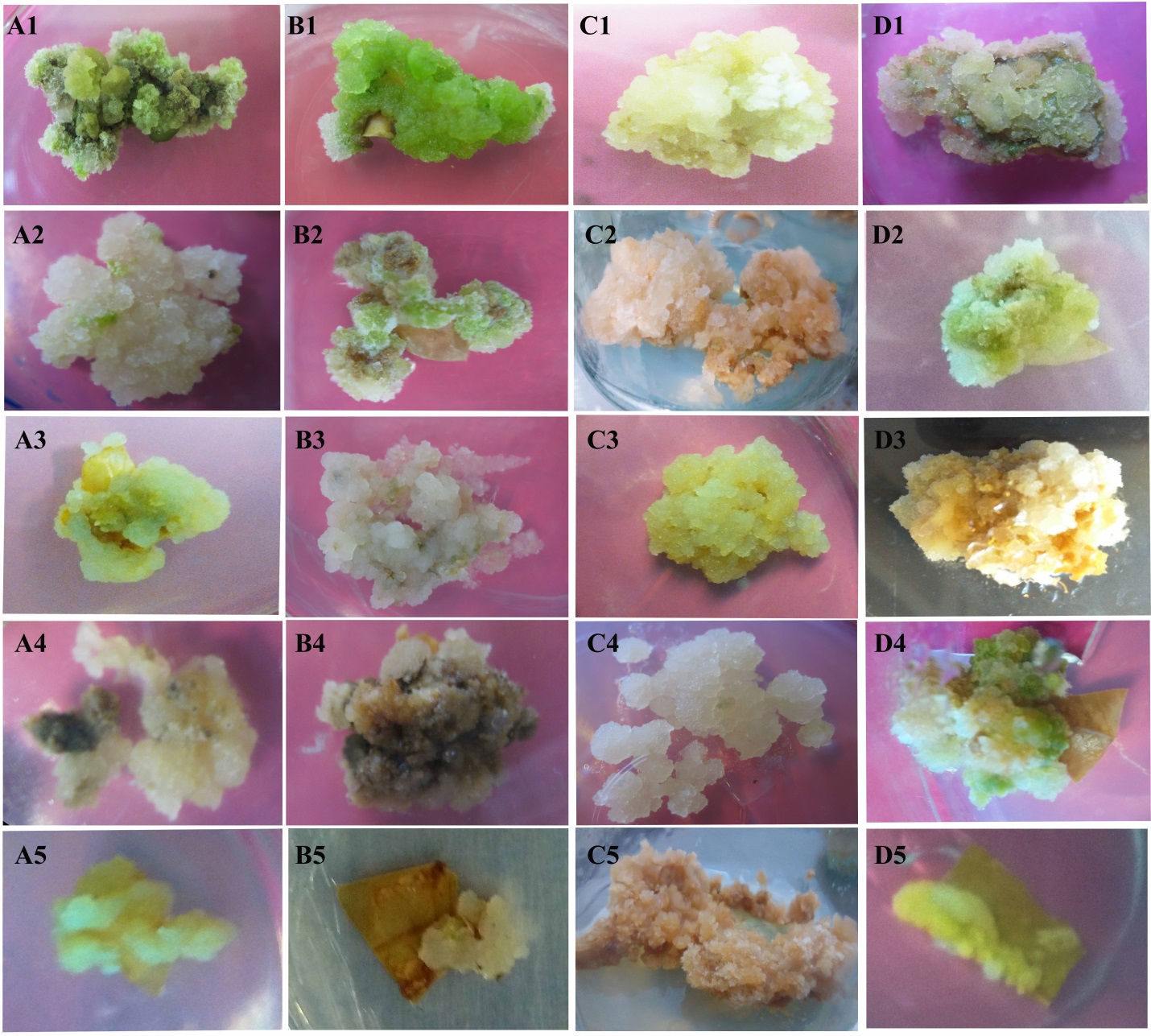
Figure S3-** *In vitro* culture of *M. azedarach* L.*;* Callus growth on MS+ A_1_: 1 mg/L NAA+1 mg/L Kin, B_1_: 1 mg/L NAA+1 mg/L BAP, C_1_: 1 mg/L 2,4-D+1 mg/L Kin, D_1_: 1 mg/L 2,4-D+1 mg/L BAP, A_2_: 3 mg/L NAA+1 mg/L Kin, B_2_: 3 mg/L NAA+1 mg/L BAP, C_2_: 3 mg/L NAA+3 mg/L Kin, D_2_: 3 mg/L NAA+3 mg/L BAP, A_3_: 3 mg/L 2,4-D+1 mg/L Kin, B_3_: 3 mg/L 2,4-D+1 mg/L BAP, C_3_: 3 mg/L 2,4-D+3 mg/L Kin, D_3_: 3 mg/L 2,4-D+3 mg/L BAP, A_4_: 5 mg/L NAA+1 mg/L Kin, B_4_: 5 mg/L NAA+1 mg/L BAP, C_4_: 5 mg/L NAA+5 mg/L Kin, D_4_: 5 mg/L NAA+5 mg/L BAP, A_5_: 5 mg/L 2,4-D+1 mg/L Kin, B_5_: 5 mg/L 2,4-D+1 mg/L BAP, C_5_: 5 mg/L 2,4-D+5 mg/L Kin, D_5_: 5 mg/L 2,4-D+5 mg/L BAP, respectively.

**
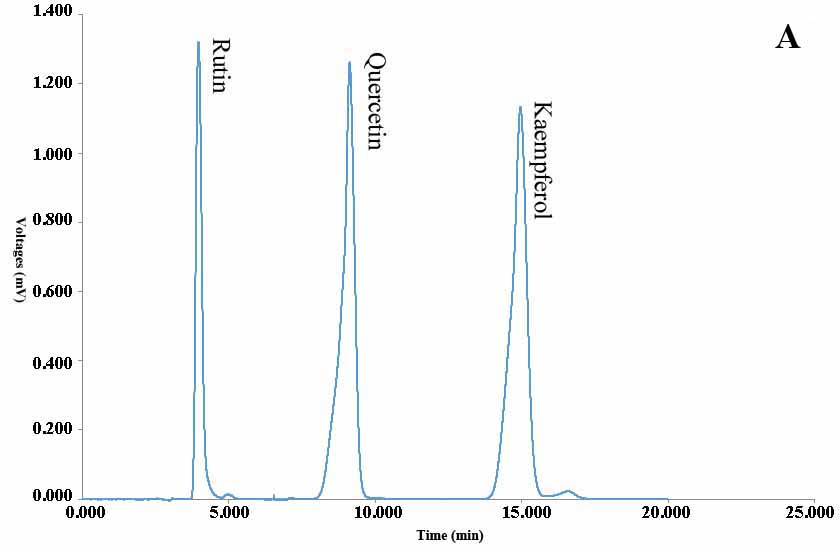

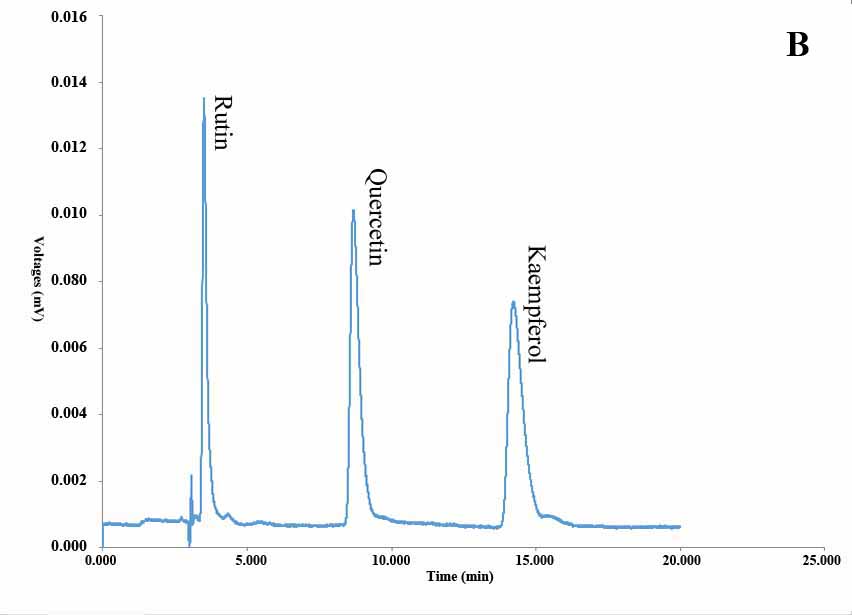
**

**Figure S4-** HPLC chromatogram of rutin, quercetin and kaempferol in the standard mixture (A) and *M. azedarach* L. calli grown on the MS medium containing 3mg/L NAA+ 3mg/L Kin (B)

**Table S1-** Different disinfection methods used in experiment 1 for sterilization of *M. azedarach* L. leaf explants

| Method | pretreatment (3 g/L Benomyl) for 2 h | Pretreatment (with 3 g/L benomyl + 0.5% NaOCl) for 2 h | 7% (v/v) H_2_O_2_ for 10 min | 5% (v/v) H_2_O_2_ for 15 min | NaOCl 2% (pH=7) for 12 min | NaOCl 2% (pH=10) for 12 min | NaOCl 2% (without pH adjustment) for 12 min |
| --- | --- | --- | --- | --- | --- | --- | --- |
| Control | - | - | - | - | - | - |  |
| A_1_ |  | - | - | - | - | - |  |
| A_2_ |  | - |  | - | - | - |  |
| A_3_ |  | - |  | - |  | - | - |
| A_4_ | - |  | - | - | - | - |  |
| A_5_ | - |  | - | - |  | - | - |
| A_6_ | - | - | - |  | - | - |  |
| A_7_ | - | - | - |  | - |  | - |

**Table S2-** Different disinfection methods and inclusion of benomyl in culture medium used in experiment 2 for sterilization of *M. azedarach* L. leaf explants

| Method | 5% H_2_O_2_ for 10 min | NaOCl 2% (pH=10) for 12 min | NaOCl 2% (without adjusted pH) for 12 min | Benomyl of medium (mg/L) |
| --- | --- | --- | --- | --- |
| Control | - | - |  | - |
| B_1_ |  | - |  | 100 |
| B_2_ |  |  | - | 100 |
| B_3_ |  | - |  | 500 |
| B_4_ |  |  | - | 500 |
| B_5_ | - | - |  | 100 |
| B_6_ | - |  | - | 100 |
| B_7_ | - | - |  | 500 |
| B_8_ | - |  | - | 500 |

**Table S3**- The effect of different disinfection methods on disinfection indices of leaf explants in *M. azedarach* L. (Experiment 1)

| S.O.V | df | MS | | | | | | | | |
| --- | --- | --- | --- | --- | --- | --- | --- | --- | --- | --- |
|  |  | Bacterial contamination  (%) | Fungal contamination  (%) | Clean explants (%) | Viability (%) | Browning (%) | Callus induction (%) | Weight of callus1 (mg/exp) | Weight of callus 2 (mg/exp) | Weight of callus 3 (mg/exp) |
| Sterilization | 7 | 0.124^**^ | 0.473^**^ | 1.026^**^ | 0.885^**^ | 0.892^**^ | 0.635^**^ | 41.441×10^3 n.s^ | 6.907×10^5 n.s^ | 61.599×10^5 **^ |
| PGR | 15 | 0.008 ^n.s^ | 0.015 ^n.s^ | 0.021 ^n.s^ | 0.054^**^ | 0.055 ^**^ | 0.057 ^*^ | 57.082×10^3 *^ | 7.677×10^5 *^ | 44.317×10^5 **^ |
| Sterilization × PGR | 105 | 0.011 ^*^ | 0.014 ^n.s^ | 0.019 ^n.s^ | 0.039^**^ | 0.041^**^ | 0.073 ^**^ | 51.834×10^3 **^ | 6.119×10^5 **^ | 24.381×10^5 **^ |
| Error | 168 | 0.008 | 0.011 | 0.022 | 0.014 | 0.014 | 0.033 | 29.209×10^3^ | 3.866×10^5^ | 10.819×10^5^ |

* and ** = significant at 0.05 and 0.01 probability level, respectively; n.s. = not significant

Weight of callus1, 2 and 3 = weight of callus (mg/explants) in the 1rd, 2rd and 3rd month after the culture, respectively.

**Table S4**- Effect of PGRs treatment on total flavonoid, total phenol and anthocyanin contents in *M. azedarach* L. calli

| S.O.V | df | Mean Square | | | |
| --- | --- | --- | --- | --- | --- |
|  |  | TFC (mg/g FW) | TPC (mg/g FW) | AC (μg/g FW) |  |
| PGR | 17 | 2.675×10^5**^ | 0.274^**^ | 1.340^**^ |  |
| Error | 36 | 0.321×10^5^ | 0.029 | 0.266 |  |
| ^**^ = Significant at the 0.01 probability level.  ^n.s.^ = not significant. | | | | | |

**Table S5**- Effect of PGRs treatment on rutin, quercetin and kaempferol in *M. azedarach* L. by HPLC analysis

| S.O.V | df | Mean Square | | |
| --- | --- | --- | --- | --- |
|  |  | Rutin (mg/g FW) | Quercetin (mg/g FW) | Kaempferol (mg/g FW) |
| PGR | 7 | 189.725^**^ | 18.722^**^ | 17.248^**^ |
| Error | 8 | 30.761 | 2.616 | 1.667 |
| ^**^ = Significant at the 0.01 probability level. | | | | |
